# Biomarker Patterns and Their Association with Lung Injury in COVID-19 Patients

**DOI:** 10.3390/medicina61050931

**Published:** 2025-05-21

**Authors:** Alexandru Constantin Sîrbu, Anca Daniela Farcaș, Ioana Corina Bocsan, Maria Adriana Neag, Ștefan Cristian Vesa, Șoimița Mihaela Suciu, Anca Dana Buzoianu

**Affiliations:** 1 Department of Pharmacology, Toxicology and Clinical Pharmacology, Iuliu Hațieganu University of Medicine and Pharmacy, 400337 Cluj-Napoca, Romania; alexandru.sirbu@umfcluj.ro (A.C.S.); bocsan.corina@umfcluj.ro (I.C.B.);; 2 Department of Internal Medicine, Cardiology and Gastroenterology, Iuliu Hațieganu University of Medicine and Pharmacy, 400006 Cluj-Napoca, Romania; 3 1st Cardiology Department, Cluj-Napoca Emergency County Hospital, 400006 Cluj-Napoca, Romania; 4 Department of Physiology, Iuliu Hațieganu University of Medicine and Pharmacy, 400006 Cluj-Napoca, Romania; mihaela.suciu@umfcluj.ro

**Keywords:** COVID-19, SARS-CoV-2, chest CT, lung, pleural effusion, pulmonary embolism, inflammatory markers

## Abstract

*Background and Objectives*: The study investigates the relationship between accessible biomarkers and the extent of lung damage, assessed with computed tomography (CT) imaging, in patients hospitalized for COVID-19. *Materials and Methods*: This retrospective analysis was conducted in a hospital in Cluj-Napoca, Romania, and it includes 111 patients diagnosed with moderate to severe forms of COVID-19 during the Delta and Omicron waves. We evaluated the association of affordable lab works, such as C-reactive protein (CRP), procalcitonin, ferritin, neutrophil and lymphocyte counts, D-dimers, and albumin levels, with the extents of lung injury, pleural effusion, pulmonary embolism, and thoracic adenopathy. *Results*: Our data show that high CRP, neutrophil counts, ferritin, and procalcitonin levels, combined with lower lymphocyte and albumin levels, were significantly associated with >25% lung damage (*p* < 0.05). Elevated ferritin (≥274 ng/mL) and neutrophil counts (≥5.2 × 10^9^/L) were independently associated with this threshold. CRP (≥2.67 mg/dL), CRP/albumin ratio (≥0.736), and ferritin had the highest sensitivity (86.8%). D-dimer was the sole biochemical marker associated with pulmonary embolism (*p* = 0.036). Pleural effusion was independently associated with lymphocyte count (cut-off < 0.605 × 10^9^/L, *p* = 0.013). Thoracic lymphadenopathy was also associated with increased neutrophil counts and a heightened inflammatory response. *Conclusions*: These findings suggest that ferritin and the CRP/albumin ratio can serve as indicators for patients with extensive parenchymal damage. D-dimer levels were the only ones significantly associated with thromboembolic events, while lymphopenia appears to be a useful indicator of pleural involvement. Thus, these readily available biomarkers can prove useful in anticipating radiological severity in patients hospitalized with COVID-19.

## 1. Introduction

The COVID-19 pandemic, caused by the SARS-CoV-2 virus, created a global health crisis, affecting millions of individuals and placing immense pressure on healthcare systems worldwide [1]. While the WHO has declared that COVID-19 is no longer a global health emergency, the disease continues to circulate globally and remains a significant public health concern [2,3]. This shift means that not only will the virus continue to cause seasonal outbreaks, like influenza and respiratory syncytial virus, but it will remain a significant health concern requiring constant attention [4].

While the clinical manifestations of COVID-19 can vary significantly from asymptomatic to critical cases with multiple organ failure, the virus is mainly considered respiratory, with the impact on the lungs being a primary concern [5]. Computed tomography (CT) imaging became an important tool in evaluating lung involvement, especially in patients with more severe forms of the disease. The characteristic findings on chest CT, including ground-glass opacities, consolidation, and signs of fibrosis, have been well-documented and are closely associated with the severity of the disease [6].

Considering the role lung damage plays in COVID-19 patients, there has been significant interest in identifying laboratory markers that correlate with imaging findings. Readily available laboratory parameters, such as leukocyte count, lymphocyte count, C-reactive protein (CRP), liver enzymes, and serum creatinine, have been studied for their prognostic value in COVID-19 [7]. Such markers are cost-effective, making them practical for routine clinical use. Ferritin, fibrinogen, and D-dimers are also considered good prognostic markers and have seen clinical use as well, especially for more severe cases [8]. The inflammatory response to SARS-CoV-2 infection plays a critical role in the pathogenesis of lung injury, with the most significant response being the cytokine storm. More particular markers, such as interleukin-6, interleukin-1, and TNF-α, play an important role in disease severity, especially in patients with acute respiratory distress syndrome (ARDS), but they are more expensive and not readily available for routine clinical use [9,10].

COVID-19 is also associated with a hypercoagulability state caused by elevated procoagulant factors, inflammation, and endothelial dysfunction, contributing to the severity of lung damage, especially in the case of pulmonary embolism [11]. D-dimer plasma levels, but also inflammation markers, such as C-reactive protein, are associated with higher frequency and severity of clotting events [12,13].

Despite the significant amount of literature on the correlation between laboratory findings and clinical outcomes in COVID-19, there is a paucity of data specifically examining the relationship between these laboratory parameters and lung damage as assessed through CT imaging. Considering the fact that COVID-19 is still an important public health issue, understanding such correlations may prove beneficial for improving risk stratification, guiding treatment, and predicting long-term outcomes in COVID-19 patients.

This retrospective study aims to explore different types of lung abnormalities, including the extent of lung injury, lymphadenopathy, pleural effusion, and pulmonary embolism on CT imaging, and their association with laboratory markers readily available for routine use. By analyzing these correlations, we hope to identify accessible and reliable biomarkers that can aid in the early detection of severe lung involvement, facilitating appropriate risk stratification and clinical intervention.

## 2. Materials and Methods

### 2.1. Study Design and Setting

We conducted a retrospective study at the County Emergency Hospital in Cluj-Napoca, Romania, a second line hospital during the height of the COVID-19 pandemic in Romania [14]. We analyzed data from adult patients with moderate to severe forms of COVID-19 during the Delta (B.1.617.2) and Omicron (B.1.1.529) waves [15] from October 2021 to May 2022 when the hospital functioned in its support capacity.

### 2.2. Participants and Variables

Inclusion criteria for the patients were as follows: a confirmed COVID-19 diagnosis based on either a positive SARS-CoV-2 rapid antigen test or a SARS-CoV-2 RT-PCR test, hospital admission between October 2021 and May 2022, age > 18 years, having at least one lung imaging evaluation performed through CT, and biochemistry tests, including protein and albumin levels, ferritin, coagulation tests, and D-dimer levels, assessed (reference ranges are included in Supplementary Table S1). For patients presenting signs of pulmonary embolism, a computed tomography pulmonary angiogram was performed. Patients with incomplete laboratory or radiological workup were excluded from the study.

COVID-19 diagnosis was established based on a positive test for the SARS-CoV-2 virus, according to the national diagnostic guidelines, either through RT-PCR or point of care antigen testing. All patients were hospitalized and received antiviral treatment, corticosteroids, anticoagulants, antibiotics, and symptomatic treatment according to the national treatment protocol.

Data were collected from the hospital’s electronic system and included demographics, clinical data, and laboratory tests performed near the point of admission, such as complete blood count, biochemistry tests, coagulation tests (prothrombin time, INR), ferritin, D-dimers, and chest CT. Lung involvement was assessed visually by a board-certified radiologist as part of their clinical practice. The extent of lung damage was visually estimated by three radiologists as a percentage of the total lung volume affected. Lung injury was defined as ground-glass opacities, crazy paving patterns, and consolidation, consistent with international CT nomenclature for COVID-19 lesions, such as those described by the Fleischner Society and other radiological guidelines [6,16,17]. Every reader made an independent visual estimate of the percentage of total lung parenchyma affected by COVID-19 (e.g., “<10%”, “≈50%”, “>75%”), the numbers were averaged, and the mean percentage was entered into the clinical report. Because there are several globally used CT severity scoring systems, radiologists provided CT severity scores (TSS or CT-SS) in some cases using either a 0–20 or 0–25 scale. However, these scoring systems were not applied uniformly, as clinicians found percentages easier to interpret than semi-quantitative scores during the pandemic. To ensure consistency, and because scoring systems are ultimately based on visually estimated lung injury, we used the radiologist-reported percentage of lung involvement in our analysis.

Patients were categorized based on the percentages of total lung surface affected: ≤25% (0–25%) lung affected by COVID-19 or >25% (26–100%) lung affected by COVID-19. This cut-off was chosen based on the previous literature, where quartile-based thresholds (e.g., 25%, 50%, 75%) are frequently used to define severity categories of COVID-19 lung abnormalities [18,19]. In particular, the 25% threshold is commonly used to distinguish mild from more significant lung injury in radiological studies. Mediastinal lymph node enlargement and pleural effusion were also evaluated.

This study was approved by the Ethics Committee of the “Iuliu Hațieganu” University of Medicine and Pharmacy, Cluj-Napoca, Romania (No. AVZ1/13.01.2025).

### 2.3. Statistical Analysis

Descriptive statistical methods were applied to the study population to outline their overall and initial characteristics, including demographic details. The Shapiro–Wilk test was used to assess the normality of quantitative variables. Data were presented using the median and the 25th and 75th percentiles. Comparisons between two independent groups concerning categorical data were performed with the chi-squared test or Fisher’s exact test. Correlation analysis was performed using Spearman’s correlation test. For comparison of two independent groups concerning normally distributed data, we used Welch’s test, and, for non-normally distributed quantitative data, Mann–Whitney tests were applied. Variables identified as statistically significant in these tests were further analyzed using receiver operating characteristic (ROC) curve while establishing cut-off points with Youden’s index. Binary logistic regression was performed to determine independent associations with the outcome. Only variables that reached *p* < 0.05 in the univariate analysis were considered for inclusion in the multivariate logistic regression after testing for multicollinearity. For variables where the initial cut-off determined by Youden’s index resulted in perfect sensitivity (100%) or specificity (100%), an alternative cut-off was selected to facilitate meaningful statistical analysis. This alternative cut-off was chosen through visual inspection of the ROC curve to balance sensitivity and specificity more evenly while maintaining clinical or practical relevance. We applied the Hosmer–Lemeshow goodness-of-fit test to check how well the binomial multivariate model matched observed outcomes. We considered *p*-values <0.05 to be statistically significant. Statistical analysis was performed using Jamovi software, version 2.3, and ROC curve analysis was performed using R (v. 4.4.1), and RStudio (v. 2024.09.0+375), with the qROC package [20,21,22,23].

## 3. Results

From October 2021 to May 2022, a total of 279 patients hospitalized with COVID-19 underwent a chest CT scan. However, only 111 of these patients had their serum protein, albumin, ferritin, and D-dimer levels evaluated. Among the population included, 80 patients underwent a native chest CT, while 21 also underwent a computed tomography pulmonary angiogram.

Therefore, our study population consisted of 111 patients hospitalized with COVID-19. Patient demographics and imaging characteristics are described in Table 1. Among the patients included, 34 had COVID-19-specific lesions affecting more than 25% of their lungs, while 73 had less than 25% of their lungs affected by the disease. Additionally, four patients had unclassifiable COVID-19 lung injuries due to overlapping conditions that made said lesions difficult to properly quantify (e.g., pulmonary embolism, fibrotic areas, and pulmonary edema). A total of 7 out of 111 patients were diagnosed with pulmonary embolism.

### 3.1. Lung Damage, Pulmonary Embolism, and Inflammatory Markers

We evaluated biochemical parameters of patients with varying degrees of lung damage due to COVID-19, classified into two groups: those with ≤25% lung damage and those with lung damage > 25%. The results are summarized in Table 2.

Patients with lung damage > 25% had significantly higher levels of CRP (9.11 vs. 2.43, *p* = 0.002), neutrophils (6.48 vs. 4.43, *p* = 0.009), procalcitonin (0.110 vs. 0.070, *p* = 0.028), and ferritin (690 vs. 241, *p* < 0.001). On the other hand, lymphocyte counts (0.850 vs. 1.14, *p* = 0.019) and serum albumin levels were significantly lower in the >25% lung damage group. Given the significant association between albumin levels and lung damage, despite not being traditionally classified as an inflammatory marker, we performed a correlation analysis between albumin and other biomarkers. Albumin showed significant inverse correlations with CRP (ρ = −0.442, *p* < 0.001), neutrophils (ρ = −0.298, *p* = 0.002), procalcitonin (ρ = −0.371, *p* < 0.001) and ferritin (ρ = −0.376, *p* < 0.001) and a positive correlation with lymphocytes (ρ = 0.296, *p* = 0.002). The correlation table with all biomarkers investigated is available in Appendix A (Table A1). We also performed a multivariate linear regression to explore which inflammatory markers were independently associated with albumin levels. CRP, neutrophils, lymphocytes, and ferritin were significantly associated, with the analysis available in Appendix A (Table A2).

Both the CRP/albumin ratio and the albumin/globulin ratio proved to be significant prognostic indicators for the extent of pulmonary lesions.

Although leukocyte counts tended to be higher in the >25% lung damage group, the difference did not reach statistical significance (8.29 vs. 6.38, *p* = 0.061). Similarly, there was a trend towards lower monocyte counts in the >25% lung damage group, and this difference was marginally non-significant (0.360 vs. 0.480, *p* = 0.053).

In Table 3, we performed a cut-off analysis based on Youden’s index for the biomarkers significantly associated with lung damage in the univariate analysis.

We performed a logistic regression (Table 4) to find which variables were independently associated with >25% lung injury due to COVID-19. Higher neutrophil counts and elevated ferritin levels were independently associated with extensive lung involvement.

In Table 5, we show our analysis of several biochemical parameters in patients with and without pulmonary embolism hospitalized with COVID-19.

D-dimer levels were significantly higher in patients with pulmonary embolism (median 748, IQR 653–1031) compared to those without (median 389, IQR 217–810), with a *p*-value of 0.036. However, the analysis showed no statistically significant differences between the two groups for other parameters.

### 3.2. Pleural Effusion in COVID-19

A detailed comparison of biological parameters in hospitalized COVID-19 patients, stratified by the presence or absence of pleural effusion, is presented in Table 6.

The analysis revealed several significant differences in biochemical parameters between patients with and without pleural effusion. Patients with pleural effusion had significantly higher CRP levels (9.80, IQR 4.44–14.3) compared to those without pleural effusion (2.71, IQR 1.46–8.25), with a *p*-value of 0.001, indicating a higher inflammatory response in patients with pleural effusion.

Although leukocyte and neutrophil counts did not differ significantly between the two groups, lymphocyte counts were significantly lower in patients with pleural effusion (0.590, IQR 0.475–1.05) compared to those without (1.08, IQR 0.818–1.44).

However, platelet counts were significantly lower in patients with pleural effusion compared to those without (190 vs. 226, *p* = 0.009), with the platelet*MPV product having a similar effect (1863 vs. 2499, *p* = 0.003).

Interestingly, prothrombin time was significantly elevated in patients with pleural effusion (PT 13.8 vs. 12.3, *p* = 0.014) and, although not statistically significant, there was a trend towards higher D-dimer levels in the pleural effusion group (median 494 vs. 385, *p* = 0.067). Protein levels were significantly lower in the pleural effusion group (median 6.04 vs. 6.42, *p* < 0.001), as were albumin levels (median 3.03 vs. 3.48, *p* < 0.001). The albumin/globulin (A/G) ratio was also lower in the pleural effusion group (median 1.05 vs. 1.16, *p* = 0.027), while the CRP/albumin (CRP/A) ratio was significantly higher in patients with pleural effusion (2.84 vs. 0.839, *p* < 0.001).

In Table 7, we show the results of our cut-off analysis based on Youden’s index for the biomarkers significantly associated with pleural effusion.

We performed a multivariate analysis (Table 8) to identify variables independently associated with pleural effusion, and lower lymphocyte count was the only one with statistical significance. The CRP/albumin ratio was excluded from the logistic regression due to multicollinearity.

### 3.3. Thoracic Adenopathy in COVID-19

Table 9 highlights the differences between patients hospitalized for COVID-19 based on the occurrence of thoracic lymphadenopathy.

Patients with lung adenopathy had significantly elevated CRP levels (4.71 vs. 2.71, *p* = 0.04) and slightly higher procalcitonin levels (0.105 vs. 0.07, *p* = 0.017), suggesting a greater inflammatory response in these patients.

Leukocyte counts tended to be higher in the lung adenopathy group, though this difference did not reach statistical significance (*p* = 0.119). However, neutrophil counts were higher in the lung adenopathy group (5.64, IQR 3.68–8.00) compared to the group without adenopathy (4.22, IQR 2.40–6.43), with a *p*-value of 0.027, suggesting an enhanced immune response.

Globulin levels were significantly higher in the lung adenopathy group (3.04, IQR 2.85–3.29) compared to those without (2.86, IQR 2.59–3.20), with a *p*-value of 0.047, indicating a shift in the protein profile. Additionally, the albumin/globulin (A/G) ratio was lower in patients with lung adenopathy compared to those without (1.10 vs. 1.19, *p* = 0.049), with a marginally significant value.

In Table 10, we show the results of the cut-off analysis based on Youden’s index for the biomarkers that showed a significant association with thoracic lymphadenopathy.

The logistic regression (Table 11) shows that elevated procalcitonin levels and prolonged prothrombin time were independently associated with thoracic adenopathy. The CRP/albumin ratio was excluded from the logistic regression analysis due to multicollinearity.

## 4. Discussion

This study investigates the relationship between common laboratory biomarkers and the extent of lung injury in patients with moderate to severe COVID-19 based on CT imaging. Our findings suggest that readily available laboratory data, including neutrophil counts, CRP, ferritin, and albumin levels, are significantly correlated with the severity of lung injury in these patients. Our analysis also revealed distinct biochemical profiles in patients with complications, such as pleural effusion, pulmonary embolism, and lung adenopathy, suggesting that these factors contribute to the inflammatory and coagulation cascades associated with severe COVID-19.

### 4.1. Inflammatory Response and Lung Damage

The physiopathology of COVID-19 has been extensively studied, with a hyper-inflammatory response, known as the cytokine storm, being one of the main causes of the disease’s severity [24]. Pneumonia, and, in more critical cases, ARDS, are the main manifestations of severe COVID-19 and result from uncontrolled inflammation in the lungs, which is why elevated levels of pro-inflammatory cytokines play a significant role in lung damage [9,25]. In order to quantify the inflammation, acute phase protein or reactants have been established as useful predictors of the disease’s clinical course [26].

In our cohort, patients with >25% lung damage had significantly higher levels of CRP, ferritin, and procalcitonin compared to those with ≤25% lung involvement. This aligns with previous research [27,28,29,30,31], which highlights that the mentioned biomarkers not only correlate with a more severe form and a poorer outcome but also with worse radiographic findings, underscoring the role of systemic inflammation in the pathogenesis of COVID-19. Based on the ROC analysis, both CRP and ferritin may be considered supportive tools to discriminate between patients based on lung involvement, with ferritin being a better option due to the independent association in multivariate analysis. While they do have moderate specificity, which limits their use as standalone indicators, their relatively high sensitivity suggests their usefulness in ruling out significant lung injury when values are low.

While albumin is traditionally considered a nutritional marker, in our cohort, it tracked closely with the acute radiological burden of disease. Inflammation increases capillary permeability, leading to the escape of serum albumin, while acute disease results in greater albumin degradation, leading to a state of hypoalbuminemia. [32,33]. In our study, serum albumin levels were significantly lower in patients with >25% lung damage (*p* = 0.021), supporting previous data suggesting that hypoalbuminemia is a potential marker of poor prognosis [34]. Furthermore, the correlation analysis we carried out revealed that lower albumin levels were significantly and inversely associated with staple inflammatory markers, namely, CRP and ferritin, and with neutrophil counts. The multivariate linear regression underscores that hypoalbuminemia in our cohort is tightly coupled to the acute inflammatory profile of COVID-19 rather than isolated biomarker changes. After simultaneous adjustment for other variables, higher concentrations of CRP and ferritin and greater neutrophil counts each remained independently associated with lower serum albumin, and higher lymphocyte count was linked to higher albumin levels. Procalcitonin lost significance in the multivariate model, indicating that its univariate association with albumin is largely explained by overlap with the stronger inflammatory predictors. Taken together, these findings confirm that albumin behaves as a negative acute-phase reactant in SARS-CoV-2 infection, not as a causative agent, but rather as a prognostic factor. Its decline reflects capillary leak, hepatic reprioritization of protein synthesis, and the general catabolic stress that accompany systemic inflammation. The CRP/albumin ratio (CRP/A) emerged as a significant marker in our study for highlighting lung involvement in COVID-19 patients. This finding aligns with the existing literature, where the CRP/A ratio has been proposed as an effective prognostic marker, with authors proposing that the ratio offers better predictability than biomarkers alone [35,36]. Similarly, based on the ROC analysis we performed, the CRP/albumin ratio actually demonstrated a slightly higher sensitivity than CRP alone (86.8% vs. 81.5%) in discriminating patients with >25% lung injury, with similar AUC and sensibility values.

Increased neutrophil counts have been identified in patients with COVID-19, and, more specifically, they have been associated with increased disease severity and a worse prognosis [31,37,38]. Data also suggest that neutrophils are present in the injured tissue, not only increased in peripheral blood [39].

Lymphopenia is another critical marker of severe COVID-19. Lymphocytes, especially T cells, are essential for controlling viral infections and modulating the immune response. In our cohort, lower lymphocyte counts were significantly associated with greater lung involvement. This finding mirrors those of previous studies, where severe cases displayed marked lymphopenia [40]. Combining readily available information, the neutrophil-to-lymphocyte ratio (NLR) has been proposed as a valuable prognostic indicator in COVID-19. Studies have shown that a high NLR is independently associated with increased mortality and a greater likelihood of complications [41,42]. Our study revealed that patients with severe lung damage (>25% lung involvement) had significantly elevated neutrophil counts (*p* = 0.009) and lower lymphocyte counts (*p* = 0.019) compared to those with less extensive lung injury. We also observed that neutrophils levels can be independent predictors of lung injury. This supports the evidence that neutrophil-driven hyperinflammation and lymphopenia are key contributors to severe lung injury in COVID-19 patients. However, our ROC analysis revealed that while neutrophil and lymphocyte counts differ significantly between patients based on lung damage, their individual ability to discriminate between these groups remains moderate. This serves as a reminder that there is a distinction between statistically significant group differences and practical classification performance.

### 4.2. Biomarkers and Coagulation Abnormalities

Our study also examined markers for pulmonary embolism and found that D-dimer levels, a marker of fibrinolysis and thrombus formation, were significantly elevated in patients with pulmonary embolism (748 vs. 389 ng/mL, *p* = 0.036). This finding is consistent with the hypercoagulable state observed in severe COVID-19, where endothelial dysfunction, inflammation, and elevated procoagulant factors contribute to thrombotic complications, such as pulmonary embolism [43,44]. Compared to other data in the literature, where inflammation markers, such as CRP and ferritin, could be predictors of pulmonary embolism [45], we identified D-dimers as the only discriminator of patients with this condition. However, we should point out that in our study, only seven patients had pulmonary embolism, and the analysis of this subgroup is underpowered. The small sample size increases the risk of a type II error, meaning that some true associations might not be properly identified as statistically significant.

### 4.3. Pleural Effusion and Lung Adenopathy

Our findings suggest that patients with pleural effusion exhibited a more pronounced inflammatory response, alterations in coagulation parameters, and reduced protein and albumin levels, highlighting the severity and complexity of their condition compared to those without pleural effusion. In our data, albumin and total protein levels demonstrated the highest discriminative ability for pleural effusion, with a relatively high sensitivity but moderate specificity. In contrast, lymphocyte count had the highest specificity and was also identified as an independent predictor of pleural effusion in our cohort, suggesting that low lymphocyte levels may be useful in evaluating the risk of pleural involvement. The incidence of pleural effusion was also higher compared to other findings in the literature in both patients with >25% lung damage (23.5%) and in those with ≤25% lung involvement (17.8%). Swenson et al. concluded in their study that pleural effusions are infrequent findings, noting that 2.1% of patients in their cohort had developed one [46]. Majidi et al. observed a higher incidence of pleural effusion at 7.6%, noting that it was a more significant event in patients over 50 years of age than in those under 50 years of age [47]. Majidi et al. also pooled data from other studies estimating a 7% pleural effusion rate. The difference in our dataset could stem not only from the severity of the cases but also from the median age of the patients being over 70 years old, supporting the data that elderly patients are more likely to develop a pleural effusion due to COVID-19.

We observed that lung adenopathy was linked to increased neutrophil counts and higher levels of CRP, further emphasizing the role of inflammation in the development of lymph node enlargement in COVID-19 patients. Data regarding thoracic lymphadenopathies in COVID-19 are heterogeneous, with some authors reporting incidences of 5% [47], while others report higher incidences ranging from 69 to 85% [48], with the highest percentage being in more severe patients.

### 4.4. Limitations of the Study

First of all, our study is limited by its observational, retrospective nature, which restricts the ability to establish clear causality between biomarkers and clinical outcomes, with unmeasured confounding factors that could influence the observed associations.

The study was conducted in a single hospital, limiting the generalizability of our results to wider patient populations. A multicenter study would provide better evidence and enhance the applicability of the findings across different patient populations and environments.

Another limitation is the timing of laboratory assessments. As we know, COVID-19 has a dynamic and heterogeneous course, and because not all patients presented on the first day of symptom onset, variations in the timing of biomarkers could affect the interpretation of acute changes in lung injury. Also, while we assessed overall lung involvement, we did not perform an analysis of individual lobes or segments in relation to the biomarkers.

Our study focused on the acute phase of COVID-19 without evaluating long-term outcomes, such as pulmonary fibrosis, pulmonary function tests, or overall survival. We also did not calculate associations. A new study with long-term follow-up data would provide valuable information on the prognostic value of the biomarkers for chronic pulmonary complications.

## 5. Conclusions

The focus of our study was the association between commonly used, easily accessible biomarkers and lung changes in COVID-19 patients, as quantified through CT imaging.

In our retrospective cohort of hospitalized patients, we observed that low-cost laboratory tests mirror the radiological burden of lung injury, as observed on chest CT. Lung involvement greater than 25% was independently associated with elevated ferritin ≥ 274 ng/mL (OR 4.9) and neutrophils ≥ 5.2 × 10^9^/L (OR 3.0). Among the biomarkers analyzed, albumin showed a correlation with lymphocyte count and an inverse correlation with the CRP, ferritin, and neutrophil count. The CRP/albumin ratio (cut-off ≥ 0.74), ferritin (cut-off ≥ 274 ng/mL), and lymphocyte count (cut-off < 0.643 10^9^/L) demonstrated the highest sensitivity (86.8%) for identifying extensive parenchymal damage. The D-dimer level was the only parameter significantly associated with pulmonary embolism. While elevated CRP levels, low lymphocyte count, and reduced albumin levels were associated with pleural effusion, only lymphocyte count (cut-off < 0.605 10^9^/L) remained an independent predictor in the multivariate analysis. Procalcitonin and prothrombin time were independently associated with thoracic lymphadenopathy, with procalcitonin demonstrating high specificity (89.8%) at a cut-off value of ≥0.2 ng/mL.

Our findings show that routinely available biomarkers can be useful in anticipating severe lung involvement and also shed light on the biochemical–radiological relationship during SARS-CoV-2 infection. Because all of these tests are available in routine biochemistry panels, they can possibly provide an inexpensive assessment when CT capacity is limited. Future research should focus on larger, multicenter cohorts to prospectively validate biomarker cut-offs and prognostic utility in relation to both acute and chronic lung injury in COVID-19.

## Figures and Tables

**Table 1:** Patient demographics and imaging characteristics.

	Lung Damage ≤ 25%	Lung Damage > 25%	Unspecified	*p*-Value *
N	73	34	4	
Male	23	17	1	0.066
Female	50	17	3	
Age (median, percentiles)	71 (64–79)	76 (63.3–82)	-	0.408
Pulmonary embolism	2	2	3	0.59
Pleural effusion	13 (17.8%)	8 (23.5%)	2	0.488
Adenopathy	32 (43.8%)	21 (61.7%)	3	0.084

* *p*-values were calculated only for patients with quantifiable lung damage (≤25% or >25%).

**Table 2:** Comparative analysis of inflammatory, hematological, and biochemical markers between two groups based on the extent of lung damage.

Parameters	Lung Damage ≤ 25%	Lung Damage > 25%	*p*-Value
N	73	34	
CRP, mg/dL	2.43 (1.24–7.40)	9.11 (2.76–13.0)	**0.002**
Leukocytes, 10^9^/L	6.38 (4.39–8.68)	8.29 (5.35–9.56)	0.061
Neutrophils, 10^9^/L	4.43 (2.90–6.90)	6.48 (4.51–7.89)	**0.009**
Platelets, 10^9^/L	204 (158–280)	229 (168–319)	0.464
MPV, fL	10.6 (9.60–11.5)	10.3 (9.60–11.1)	0.652 *
Platelet × MPV	2183 (1711–2823)	2551 (1675–2956)	0.46
Monocytes, 10^9^/L	0.480 (0.350–0.660)	0.360 (0.263–0.552)	0.053
Lymphocytes, 10^9^/L	1.14 (0.740–1.50)	0.850 (0.593–1.16)	**0.019**
Procalcitonin, ng/mL	0.070 (0.040–0.160)	0.110 (0.0675–0.255)	**0.028**
D-dimers, ng/mL	371 (216–891)	420 (330–651)	0.766
Prothrombin time, s	12.4 (11.4–14.3)	12.9 (11.9–14.0)	0.561
INR	1.10 (1.01–1.20)	1.12 (1.05–1.25)	0.297
Proteins, g/dL	6.30 (5.97–6.81)	6.36 (6.01–6.60)	0.521 *
Albumin, g/dL	3.41 (3.04–3.85)	3.25 (3.06–3.48)	0.021 *
Globulin, g/dL	2.88 (2.60–3.22)	3.09 (2.80–3.20)	0.310 *
A/G (albumin/globulin ratio)	1.20 (1.02–1.34)	1.05 (1.00–1.16)	**0.022**
CRP/A (CRP/albumin ratio)	0.723 (0.308–2.39)	2.74 (0.881–4.03)	**0.001**
Urea, mg/dL	46 (36.0–72.0)	53.5 (37.8–67.3)	0.497
Creatinine, mg/dL	0.830 (0.630–1.06)	0.815 (0.623–1.07)	0.904
Uric acid, mg/dL	5.29 (4.08–6.99)	5.57 (4.38–7.65)	0.747
Ferritin, ng/mL	241 (131–655)	690 (402–1302)	**<0.001**

* Welch’s test; CRP—C-reactive protein, MPV—mean platelet volume, INR—International Normalized Ratio.

**Table 3:** Biomarker cut-off analysis for the extent of lung damage.

Variables	Cut-Off Value	AUC (95% CI)	Sensitivity	Specificity
CRP	2.67 mg/dL	0.693 (0.591–0.795)	81.5% (68.2–92.1)	54.7% (42.4–65.7)
Neutrophils	5.215 10^9^/L	0.666 (0.558–0.774)	68.4% (55.2–81.5)	67.1% (56.1–78.0)
Lymphocytes	1.275 10^9^/L	0.643 (0.534–0.751)	86.8% (73.6–97.3)	41.1% (30.1–52.0)
Procalcitonin	0.185 ng/mL	0.617 (0.506–0.727)	38.8% (25.0–55.5)	81.1% (71.0–89.8)
Albumin	3.505 g/dL	0.615 (0.512–0.718)	81.5% (68.4–92.1)	46.6% (35.6–57.5)
Ferritin	274 ng/mL	0.727 (0.631–0.824)	86.8% (76.3–97.4)	54.8% (43.8–65.8)
A/G	1.177	0.648 (0.545–0.752)	78.9% (65.8–92.1)	56.2% (43.8–67.1)
CRP/A	0.736	0.694 (0.592–0.796)	86.8% (76.3–97.3)	52.0% (41.0–63.0)

**Table 4:** Multivariate analysis based on the extent of lung damage.

Variables	Estimate	*p*	OR	95% C.I for OR	
				Min	Max
CRP	0.205	0.847	1.227	0.1543	9.76
**Neutrophils**	**1.104**	**0.026**	**3.016**	**1.1384**	**7.99**
Lymphocytes	−1.078	0.101	0.34	0.094	1.23
Procalcitonin	0.348	0.542	1.416	0.4625	4.34
Albumin	−0.235	0.701	0.79	0.2381	2.62
**Ferritin**	**1.589**	**0.009**	**4.899**	**1.5001**	**16**
A/G	−0.746	0.204	0.474	0.1501	1.5
CRP/A	0.654	0.577	1.923	0.1938	19.08

Goodness-of-fit: Hosmer–Lemeshow (df = 8), *p* = 0.63.

**Table 5:** Comparative analysis of inflammatory, hematological, and biochemical markers between two groups based on the occurrence of pulmonary embolism.

Pulmonary Embolism	Yes	No	*p*-Value
N	7	104	
CRP, mg/dL	4.89 (3.39–13.7)	3.30 (1.51–9.53)	0.205
Leukocytes, 10^9^/L	6.07 (4.73–8.24)	6.62 (4.63–9.40)	0.799
Neutrophils, 10^9^/L	4.82 (3.16–6.67)	4.92 (3.23–7.60)	1
Platelets, 10^9^/L	213 (203–275)	205 (159–309)	0.804
MPV, fL	10.4 (9.65–11.7)	10.4 (9.60–11.3)	0.616 *
Platelet × MPV	2556 (2185–2927)	2210 (1678–2933)	0.606
Monocytes, 10^9^/L	0.350 (0.265–0.525)	0.460 (0.310–0.590)	0.347
Lymphocytes, 10^9^/L	0.960 (0.675–1.23)	1.00 (0.665–1.38)	0.602
Procalcitonin, ng/mL	0.0600 (0.0500–0.0700)	0.0800 (0.0500–0.1900)	0.41
D-dimers, ng/mL	748 (653–1031)	389 (217–810)	**0.036**
Prothrombin time, s	13.8 (13.3–14.2)	12.6 (11.6–14.1)	0.224
INR	1.23 (1.13–1.26)	1.10 (1.02–1.20)	0.194
Proteins, g/dL	6.22 (5.94–6.71)	6.32 (6.00–6.76)	0.827 *
Albumin, g/dL	3.14 (3.13–3.46)	3.39 (3.04–3.69)	0.759 *
Globulin, g/dL	3.08 (2.85–3.28)	2.95 (2.62–3.22)	0.425 *
A/G (albumin/globulin ratio)	1.08 (1.03–1.17)	1.13 (0.993–1.31)	0.441
CRP/A (CRP/albumin ratio)	1.54 (1.08–4.10)	0.972 (0.416–2.92)	0.227
Urea, mg/dL	37 (33.0–56.5)	48.0 (36.0–69.0)	0.347
Creatinine, mg/dL	0.740 (0.610–0.945)	0.825 (0.625–1.06)	0.693
Uric acid, mg/dL	7.63 (6.46–7.72)	5.28 (4.08–7.37)	0.179
Ferritin, ng/mL	277 (85.5–347)	404 (186–842)	0.084

* Welch’s test; CRP—C-reactive protein, MPV—mean platelet volume, INR—International Normalized Ratio.

**Table 6:** Comparative analysis of inflammatory, hematological, and biochemical markers between two groups based on the occurrence of pleural effusion.

Pleural Effusion	Yes	No	*p*-Value
N	23	88	
CRP, mg/dL	9.80 (4.44–14.3)	2.71 (1.46–8.25)	**0.001**
Leukocytes, 10^9^/L	6.54 (3.43–10.0)	6.67 (4.65–8.90)	0.819
Neutrophils, 10^9^/L	5.26 (3.05–8.10)	4.83 (3.32–7.48)	0.634
Platelets, 10^9^/L	190 (137–211)	226 (168–321)	**0.009**
MPV, fL	10.3 (9.60–11.2)	10.4 (9.60–11.3)	0.829 *
Platelet × MPV	1863 (1430–2380)	2499 (1829–2992)	**0.003**
Monocytes, 10^9^/L	0.350 (0.290–0.585)	0.475 (0.320–0.575)	0.313
Lymphocytes, 10^9^/L	0.590 (0.475–1.05)	1.08 (0.818–1.44)	**<0.001**
Procalcitonin, ng/mL	0.140 (0.085–0.385)	0.0700 (0.0425–0.155)	**0.004**
D-dimers, ng/mL	494 (356–1200)	385 (215–779)	0.067
Prothrombin time, s	13.8 (12.8–15.4)	12.3 (11.4–13.9)	**0.014**
INR	1.20 (1.10–1.30)	1.08 (1.01–1.20)	0.015
Proteins, g/dL	6.04 (5.54–6.31)	6.42 (6.11–6.89)	<0.001 *
Albumin, g/dL	3.03 (2.60–3.25)	3.48 (3.10–3.77)	<0.001 *
Globulin, g/dL	2.88 (2.59–3.19)	2.99 (2.66–3.24)	0.478 *
A/G (albumin/globulin ratio)	1.05 (0.910–1.16)	1.16 (1.02–1.32)	**0.027**
CRP/A (CRP/albumin ratio)	2.84 (1.37–4.93)	0.839 (0.373–2.61)	**<0.001**
Urea, mg/dL	60 (40.5–78.0)	46.0 (35.8–65.3)	0.079
Creatinine, mg/dL	0.910 (0.735–1.19)	0.790 (0.610–0.955)	0.076
Uric acid, mg/dL	6.05 (3.77–7.70)	5.24 (4.33–7.11)	0.764
Ferritin, ng/mL	406 (259–812)	352 (162–807)	0.469

* Welch’s test; CRP—C-reactive protein, MPV—mean platelet volume, INR—International Normalized Ratio.

**Table 7:** Biomarker cut-off analysis for pleural effusion.

Variables	Cut-Off Value	AUC (95% CI)	Sensitivity	Specificity
CRP	3.71 mg/dL	0.716 (0.592–0.841)	82.6% (65.2–95.7)	59.1% (48.9–69.3)
Platelets	213.5 10^9^/L	0.677 (0.564–0.791)	78.3% (60.9–95.7)	53.4% (43.2–63.6)
Platelet × MPV	2496.5	0.702 (0.586–0.818)	82.6% (65.2–95.7)	51.1% (40.9–61.4)
Lymphocytes	0.605 10^9^/L	0.731 (0.605–0.857)	56.5% (34.8–78.3)	86.4% (78.4–93.2)
Procalcitonin	0.105 ng/mL	0.697 (0.575–0.819)	73.9% (56.4–91.3)	68.3% (58.5–78.1)
Prothrombin Time	12.85 s	0.672 (0.556–0.788)	72.7% (54.6–90.9)	61.5% (51.8–72.3)
Proteins	6.39 g/dL	0.748 (0.648–0.847)	82.6% (61.2–95.0)	51.1% (40.0–61.9)
Albumin	3.32 g/dL	0.793 (0.704–0.882)	86.9% (66.4–97.2)	59.0% (48.1–69.5)

**Table 8:** Multivariate analysis based on the occurrence of pleural effusion.

Variables	Estimate	*p*	OR	95% C.I for OR	
				Min	Max
CRP	1.171	0.161	3.224	0.6264	16.597
Platelets	−0.487	0.683	0.614	0.059	6.395
Platelet × MPV	−1.655	0.185	0.191	0.0166	2.205
**Lymphocytes**	**−1.735**	**0.013**	**0.176**	**0.0448**	**0.695**
Procalcitonin	1.093	0.113	2.984	0.7713	11.548
Prothrombin Time	0.655	0.334	1.925	0.5104	7.259
Total Proteins	−1.542	0.095	0.214	0.035	1.309
Albumin	−0.799	0.406	0.45	0.0683	2.961
A/G	−0.461	0.588	0.631	0.1191	3.342

Goodness-of-fit: Hosmer–Lemeshow (df = 8), *p* = 0.18.

**Table 9:** Comparative analysis of inflammatory, hematological, and biochemical markers between two groups based on the occurrence of thoracic lymphadenopathy.

Thoracic Adenopathy	Yes	No	*p*-Value
N	56	55	
CRP, mg/dL	4.71 (2.02–10.7)	2.71 (1.32–7.44)	**0.04**
Leukocytes, 10^9^/L	7.90 (4.93–9.75)	6.28 (4.21–8.25)	0.119
Neutrophils, 10^9^/L	5.64 (3.68–8.00)	4.22 (2.40–6.43)	**0.027**
Platelets, 10^9^/L	214 (166–321)	201 (159–277)	0.585
MPV, fL	10.4 (9.60–11.3)	10.5 (9.60–11.4)	0.956 *
Platelet × MPV	2392 (1707–2951)	2180 (1685–2916)	0.73
Monocytes, 10^9^/L	0.475 (0.300–0.615)	0.430 (0.325–0.560)	0.697
Lymphocytes, 10^9^/L	0.925 (0.597–1.29)	1.02 (0.785–1.38)	0.213
Procalcitonin, ng/mL	0.105 (0.0500–0.255)	0.0700 (0.0400–0.120)	**0.017**
D-dimers, ng/mL	447 (276–834)	374 (213–854)	0.559
Prothrombin time, s	12.6 (11.9–14.0)	12.8 (11.4–14.7)	0.827
INR	1.10 (1.02–1.21)	1.10 (1.02–1.27)	0.729
Proteins, g/dL	6.32 (6.08–6.71)	6.30 (5.84–6.80)	0.49 *
Albumin, g/dL	3.30 (3.01–3.62)	3.40 (3.09–3.73)	0.331 *
Globulin, g/dL	3.04 (2.85–3.29)	2.86 (2.59–3.20)	0.047 *
A/G (albumin/globulin ratio)	1.10 (0.956–1.25)	1.19 (1.03–1.36)	**0.049**
CRP/A (CRP/albumin ratio)	1.70 (0.618–3.73)	0.886 (0.352–2.05)	**0.04**
Urea, mg/dL	52.0 (37.0–72.0)	45 (36.0–65.5)	0.282
Creatinine, mg/dL	0.830 (0.625–1.10)	0.800 (0.63–0.985)	0.497
Uric acid, mg/dL	5.28 (4.35–7.69)	5.32 (3.86–7.17)	0.692
Ferritin, ng/mL	412 (190–875)	350 (165–799)	0.511

* Welch’s test; CRP—C-reactive protein, MPV—mean platelet volume, INR—International Normalized Ratio.

**Table 10:** Biomarker cut-off analysis for thoracic lymphadenopathy.

Variables	Cut-Off Value	AUC (95% CI)	Sensitivity	Specificity
CRP	8.58 mg/dL	0.613 (0.508–0.718)	41.1 (28.6–53.6)	81.8 (70.9–90.9)
Neutrophils	6.11 10^9^/L	0.622 (0.516–0.727)	48.2 (35.7–60.7)	74.6 (61.8–85.5)
Procalcitonin	0.2 ng/mL	0.635 (0.530–0.741)	35.7 (23.2–48.2)	89.8 (81.6–98.0)
Globulin	2.865 g/dL	0.604 (0.498–0.711)	75.0 (62.5–85.7)	50.9 (38.2–63.6)
A/G Ratio	1.144	0.609 (0.504–0.714)	62.5 (50.0–75.0)	56.4 (43.6–69.1)

**Table 11:** Multivariate analysis based on the occurrence of thoracic lymphadenopathy.

Variables	Estimate	*p*	OR	95% C.I for OR	
				Min	Max
CRP	0.537	0.324	1.7106	0.5883	4.974
Neutrophils	0.596	0.206	1.8146	0.7205	4.57
**Procalcitonin**	**1.47**	**0.015**	**4.3491**	**1.3327**	**14.193**
**Prothrombin Time**	**1.183**	**0.022**	**3.265**	**1.1857**	**8.99**
Globulin	0.392	0.457	1.4807	0.5261	4.168
A/G	0.537	0.324	1.7106	0.5883	4.974

Goodness-of-fit: Hosmer–Lemeshow (df = 6—due to sample size), *p* = 0.81.

## Data Availability

The datasets presented in this article are not readily available because the data are part of an ongoing study.

## Supplementary Materials

Reference ranges for the biomarkers used can be downloaded at https://www.mdpi.com/article/10.3390/medicina61050931/s1, Table S1: Reference ranges for adults for biomarkers used in the study.

## Appendix A

**Table A1:** Spearman’s correlation between seric albumin and biological markers.

Parameter	Spearman’s Rho	*p*-Value
CRP	−0.442	<0.001
Leukocytes	−0.212	0.028
Neutrophils	−0.298	0.002
Platelets	−0.048	0.621
MPV	0.178	0.067
Monocytes	−0.003	0.974
Lymphocytes	0.296	0.002
Procalcitonin	−0.371	<0.001
D-Dimers	−0.426	<0.001
Prothrombin Time	−0.24	0.016
INR	−0.235	0.018
Urea	−0.169	0.082
Creatinine	−0.03	0.756
Uric Acid	0.077	0.451
Ferritin	−0.376	<0.001

**Table A2:** Multivariate linear regression analysis of inflamatory markers based on albumin levels.

Parameter	Estimate	*p*-Value
**CRP**	**−0.01999**	**0.023**
**Neutrophils**	**−0.03533**	**0.018**
**Lymphocytes**	**0.1442**	**0.036**
Procalcitonin	−0.00195	0.649
**Ferritin**	**−0.00014**	**0.03**
