# Supplementary material for: Biomarker Patterns and Their Association with Lung Injury in COVID-19 Patients
Source: Medicina (Kaunas). 2025 May 21;61(5):931. doi: 10.3390/medicina61050931 (PMC12113636; doi:10.3390/medicina61050931)
Supplement: Supplementary file 1 [file medicina-61-00931-s001.zip › medicina-3602874-supplementary.pdf]

Table S1. Reference ranges for adults for biomarkers used in the study

Parameters	**Reference range**
CRP	<0.5 mg/dl
Leukocytes	4-10 (10^9/L)
Neutrophils	1.5-6.6 (10^9/L)
Platelets	150-400 (10^9/L)
MPV	6.5-12 fL
Monocytes	0.21-0.92 (10^9/L)
Lymphocytes	1.1-3.5 (10^9/L)
Procalcitonin	<0.06 ng/ml
D-dimers	<243 ng/ml
Prothrombin Time	9-13 seconds
INR	0.79-1.16
Proteins	6.6-8.3 g/dl
Albumin	3.5-5.2 g/dl
Urea	17-43 mg/dl
Creatinine	0.51-0.95 mg/dl
Uric Acid	2.6-6 mg/dl
Ferritin	10-120 ng/ml
